# Transovarial Transmission of *Borrelia hermsii* by Its Tick Vector and Reservoir Host *Ornithodoros hermsi*

**DOI:** 10.3390/microorganisms9091978

**Published:** 2021-09-17

**Authors:** Tom G. Schwan, Sandra J. Raffel

**Affiliations:** Laboratory of Bacteriology, Rocky Mountain Laboratories, National Institute of Allergy and Infectious Diseases, National Institutes of Health, 903 South 4th Street, Hamilton, MT 59840, USA; sstewart@nih.gov

**Keywords:** relapsing fever spirochetes, borreliosis, zoonoses, soft ticks, Argasidae

## Abstract

Transovarial passage of relapsing fever spirochetes (*Borrelia* species) by infected female argasid ticks to their progeny is a widespread phenomenon. Yet this form of vertical inheritance has been considered rare for the North American tick *Ornithodoros hermsi* infected with *Borrelia hermsii*. A laboratory colony of *O. hermsi* was established from a single infected female and two infected males that produced a population of ticks with a high prevalence of transovarial transmission based on infection assays of single and pooled ticks feeding on mice and immunofluorescence microscopy of eggs and larvae. Thirty-eight of forty-five (84.4%) larval cohorts (groups of larvae originating from the same egg clutch) transmitted *B. hermsii* to mice over four and a half years, and one hundred and three single and one hundred and fifty-three pooled nymphal and adult ticks transmitted spirochetes during two hundred and fourteen of two hundred and fifty-six (83.6%) feedings on mice over seven and a half years. The perpetuation of *B. hermsii* for many years by infected ticks only (without acquisition of spirochetes from vertebrate hosts) demonstrates the reservoir competence of *O. hermsi*. *B. hermsii* produced the variable tick protein in eggs and unfed larvae infected by transovarial transmission, leading to speculation of the possible steps in the evolution of borreliae from a tick-borne symbiont to a tick-transmitted parasite of vertebrates.

## 1. Introduction

Theobald Smith and Frederick Kilborne [1] were first to show that a blood-feeding arthropod was the biological vector of a pathogen (in their case, the protozoan cause of Texas cattle fever). In a series of beautifully designed field experiments, they demonstrated that the tick, *Rhipicephalus (Boophilus) annulatus* (Say) [=*Boophilus bovis* (Riley)], was required for the horizontal transfer of *Babesia bigemina* from infected to naïve cattle. An essential link in this transmission cycle was a new generation of larval ticks that acquired their parasites from the infected females that gave birth to them. This discovery was the first demonstration of vertical passage of a pathogen from infected female ticks to their progeny.

In the first description of a tick-borne disease of humans (relapsing fever in Africa) the spirochetal cause *Borrelia duttonii* was also shown to be passed to the next generation of ticks by females of its vector *Ornithodoros moubata* [2] via infected eggs [3,4]. In the 50 years that followed, the ability of other species of argasid ticks, primarily species of *Ornithodoros*, to transovarially pass spirochetes to their progeny became known [5,6,7]. The most recent review of the *Borrelia* species associated with the relapsing fever group of spirochetes found that 11 of the 26 species discussed may be transmitted transovarially by their respective tick vectors [8], and the other species not yet studied may do so as well.

*Borrelia hermsii* is the primary cause of relapsing fever associated with argasid ticks in the United States [9] and is maintained in enzootic foci by its specific tick vector *Ornithodoros hermsi* and small mammals [10,11]. Few studies have addressed transovarial transmission of *B. hermsii* by *O. hermsi* and this form of vertical transmission has been considered rare for this spirochete by its arthropod host [12,13,14]. During an investigation of a human case of relapsing fever at Mt. Wilson Observatory in the San Gabriel Mountains, Los Angeles County, California, nine *O. hermsi* nymphs were collected [15]. Six ticks were tested in a single group by PCR that was infected with *B. hermsii*. Three additional nymphs each transmitted *B. hermsii* when fed on mice in the laboratory. Subsequently, these infected nymphs developed into adults yielding one female and two males. A colony of ticks was established from these founders described herein that displayed a high prevalence of transovarial transmission that contrasted with the few previous observations regarding this phenomenon for *O. hermsi* infected with *B. hermsii*.

## 2. Materials and Methods

### 2.1. Bacterial Strain

The *B. hermsii* strain MTW-4 observed herein was isolated previously from the blood of a mouse infected by the *O. hermsi* nymph collected at Mt. Wilson Observatory that developed into the founding female of the colony. Multilocus sequence typing of the isolate identified the spirochete as *B. hermsii* belonging to Genomic Group II [15]. For the work presented herein, the spirochetes in the female tick were acquired transtadially from its preceding naturally infected nymph and were not manipulated in vitro prior to establishing the colony.

### 2.2. Tick Feeding and Colony Maintenance

The *O. hermsi* colony was established at the Rocky Mountain Laboratories (RML) and originated from the three nymphs collected at Mt. Wilson Observatory [15] as mentioned above. Once these ticks developed into adults, the feeding and fecundity of the single female paired with the two males was tracked individually when fed on mice, however, not all of the female’s feedings were followed for transmission. As the colony expanded, ticks were held in numbered tubes to follow their lineage. Ticks were fed individually or in pools to assess their infection by transmission to mice. Other feedings were done only to maintain and expand the colony and are not included herein. Ticks were fed on laboratory mice, *Mus musculus*, five to seven days old (pups) from litters of various sizes. Single pups were placed in a plastic, round, clear jar (7 cm high, 5.5 cm diameter) with a screw-capped lid perforated in the middle with a 1.5 cm diameter opening covered with nylon mesh screen, and a 1 cm thick plaster-of-Paris lining on the bottom [16]. Single or pooled ticks of various numbers were put in the chambers and placed in the dark for 1 h at room temperature. Tubes containing new larval cohorts (larvae originating from the same clutch of eggs) were first chilled with crushed ice to immobilize and separate them from the adults before placing the larvae in feeding chambers. After feeding, the pups were removed from the chambers, brushed while being examined with a stereoscopic dissecting microscope to remove any ticks, marked individually with ink on their tail or head, and returned to their cage and dam. Mice used only for colony maintenance were euthanized by inhalation of isoflurane (Fluriso; Vet ONE, MWI Veterinary Supply, Boise, ID, USA) immediately after the ticks fed. Engorged ticks were kept overnight in the feeding chambers to allow them to excrete coxal fluid on the absorbent plaster [17] before they were counted as fed or unfed and returned to their holding tubes. Cohorts of engorged larvae were placed in new numbered tubes. Ticks were kept between feedings at room temperature (~21 °C) and ambient light in 15- or 50-mL plastic Corning tubes containing strips of filter paper to absorb moisture and perforated caps for ventilation. The tubes were kept inside closed, glass hydrating jars with a bottom reservoir containing a saturated KCl solution that provided a relative humidity of 85%.

### 2.3. Spirochete Infection in Mice

**Figure 1:**
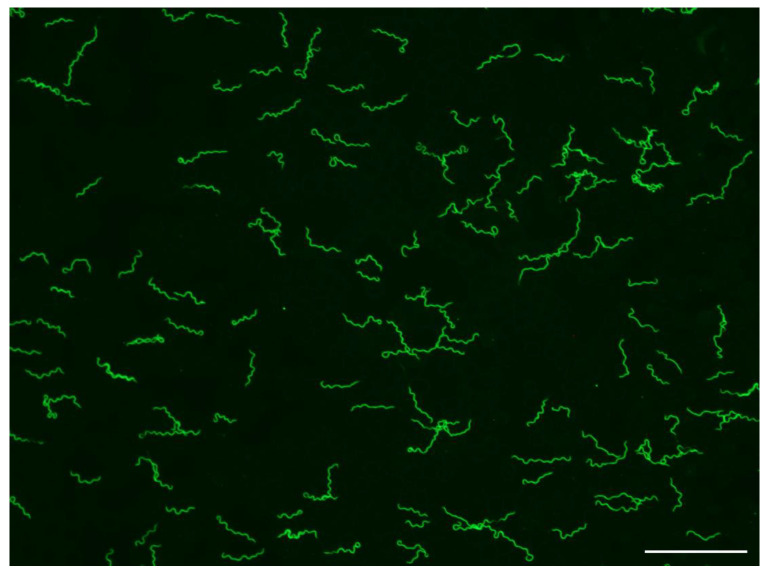
*Borrelia hermsii* MTW-4 in a thin, methanol-fixed mouse blood smear stained with anti-*B. hermsii* rabbit hyperimmune serum and goat anti-rabbit FITC. Scale bar = 40 µm.

Mice used to assess tick infection by transmission were examined for spirochetemias starting day three after tick feeding. Mice were anesthetized by inhalation of isoflurane, the tail vein was pricked with a sterile lancet, a drop of blood was expressed onto a glass microscope slide, covered with a 22-mm^2^ glass coverslip, and examined at 400× with a Nikon Eclipse E600 dark-field microscope (Nikon Instruments, Melville, NY, USA) using a 40× dry objective lens. Most infected mice had detectable spirochetemias beginning days three to five after ticks had fed on them and were euthanized. Those pups not showing a spirochetemia after examining 50 fields were returned to their dam and checked for infection until day 8 when the examinations ended. However, the MTW-4 strain of *B. hermsii* infecting the ticks produced high spirochetemias in the mice (Figure 1), such that early in their infection, an examination of only one or two microscopic fields of blood was required to detect spirochetes.

### 2.4. Spirochete Infection in Eggs and Larvae

**Figure 2:**
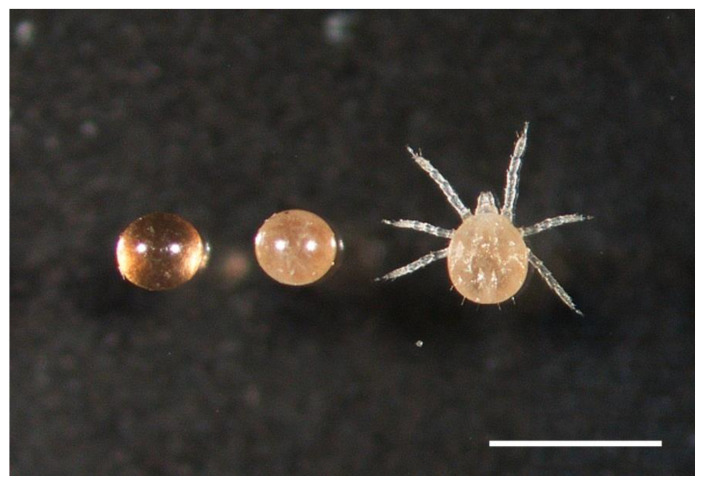
*Ornithorodos hermsi* unembryonated egg (**left**), embryonated egg (**middle**) and unfed larva (**right**). Scale bar = 1.0 mm.

Eggs and larvae (Figure 2) were prepared individually to examine them for spirochetes by rupturing their contents in a drop of phosphate-buffered saline (PBS) on a glass microscope slide, discarding the shell or exoskeleton, drying at room temperature, and fixing with heat and acetone as described for other tick tissues [18]. Spirochetes were stained with a rabbit anti-*B. hermsii* hyper-immune serum #2779 diluted 1:50 in PBS and goat anti-rabbit IgG (H & L chain) antibody conjugated with fluorescein isothiocyanate (FITC) (Kirkegaard & Perry, Gaithersburg, MD, USA) diluted 1:100 in PBS. Additional eggs and larvae were stained with anti-variable tick protein (Vtp) Type 5-specific mouse monoclonal antibody H3548 [19] and goat anti-mouse IgG (H & L chain) antibody conjugated with rhodamine isothiocyanate (RITC) (Kirkegaard & Perry) to assess if spirochetes were producing Vtp [19]. This monoclonal antibody is specific to *B. hermsii* and only those strains producing Vtp Type 5 [19]. The samples were examined for spirochetes with a Nikon Eclipse E800 epifluorescence microscope (Nikon Instruments) at 600× using a 60× oil immersion objective lens. 

### 2.5. Examination of Unfed Larvae by Dark-Field Microscopy

On occasions when larvae did not feed with their cohort, they were teased apart in a drop of PBS on a microscope slide and examined for spirochetes with a Nikon Eclipse E600 dark-field microscope at 400× using a 40× dry objective lens. 

### 2.6. Experimental Infection and Transmission of a Genomic Group I B. hermsii

When the first observations were made showing transovarial transmission of *B. hermsii* MTW-4 (Genomic Group II) by the founding female tick from Mt. Wilson, an experimental infection was performed to address if transovarial transmission could occur with a different lineage of ticks and strain of *B. hermsii*. A BSK-II culture of *B. hermsii* DAH, a clinical isolate from eastern Washington belonging to Genomic Group I [20], was inoculated intraperitonealy into an adult laboratory mouse. Four days later, 20 adult *O. hermsi* from a clean colony (SIS) that originated with ticks from Siskiyou County, California, were fed on the spirochetemic mouse and maintained as described for the MTW colony. Four months later, approximately 400 larvae from 13 *O. hermsi* females were fed on 5-day old mice as described and monitored daily for spirochetemias by examining a drop of blood from the tail vein by dark-field microscopy.

### 2.7. Statistical Analyses

The prevalence of infections for some results include 95% confidence intervals (CI) and differences in the number of infections between groups are compared with the Chi square test (*X*^2^).

### 2.8. Ethics Statement

The RML, NIAID, NIH, Animal Care and Use Committee approved study protocols for feeding ticks on mice (2009-87, 2012-70, 2015-82, 2018-062) and infecting and sampling mice for spirochetemias (2009-32, 2012-29, 2015-084). All work was done in adherence to the institution’s guidelines for animal husbandry and followed the guidelines and basic principals in the Public Health Service Policy on Humane Care and Use of Laboratory Animals, and the Guide for the Care and Use of Laboratory Animals, United States Institute of Laboratory Animal Resources, National Research Council.

## 3. Results

### 3.1. Fecundity and Transovarial Transmission by the Founding Female

The founding *O. hermsi* female fed nine times as an adult during four years (Table 1) before it was found dead in September 2013. After each of its first seven bloodmeals, the tick produced a clutch of eggs that yielded infected offspring determined by feeding larvae and/or nymphs on mice, and in a few cases supported by examination of unfed larvae by dark-field microscopy. As the tick aged, its fecundity decreased as it produced only eight larvae after the seventh feeding (18–46 larvae for all previous feedings) and none following its eighth and ninth bloodmeals, when the tick also did not cause infection in mice it fed upon. However, during its life this tick contributed 189 larvae to the colony that fed and likely had a high prevalence of spirochete infection by transovarial transmission (see below).

### 3.2. Prevalence of Spirochete Infection in F-1 Adults and Transovarial Transmission

All seven clutches produced by the founding female had progeny infected transovarially, although the prevalence of infection among individual ticks was not known. Therefore, as first generation (F-1) adults developed from the first two clutches produced by the founding female, some of these ticks were fed individually on mice and were followed for infection. In all, 36 of 39 F-1 adults (92.3%; 95% CI, 83.9–100%) (18 of 20 males, 18 of 19 females) produced infections in mice upon which they fed. The F-1 females were paired with F-1 males after their first bloodmeal and followed for their production of infected offspring (Table 2). While 18 of 19 females were infected based on transmission to mice, the one female (F-1G) that did not transmit was also infected as it produced two cohorts of infected progeny. Thus, the prevalence of infection in these 19 F-1 females was 100%.

The majority of these F-1 females (16 of 19; 84.2%) produced one or more cohorts of larvae infected transovarially following their first and subsequent bloodmeals (Table 2). While most of the larval cohorts were infected, three infected females, F-1B, F-1K, F-1P, each produced one of their larval cohorts that was noninfective to mice (Table 2). The other exceptions were two infected females, F-1L and F-1O, that each produced two larval cohorts that failed to infect mice they fed upon. One infected female, F-1H, never oviposited. Overall, the 18 F-1 females that reproduced (all infected by transovarial and transtadial passage of *B. hermsii* from the founding female) produced 40 cohorts of larvae, of which 33 (82.5%; 95% CI, 70.7–94.3%) were infective to mice. Seven of these F-1 females (F-1B–F-1G, F-1I) produced larval cohorts that infected mice 2.9 to 3.7 years after their parental female’s first bloodmeal. These observations demonstrate the prolonged and consistent ability of these ticks to transovarially infect their offspring, as did the founding female of the colony (Table 1).

### 3.3. Prevalence of Larval Cohorts Infected by Transovarial Transmission as the Colony Aged

Forty-five cohorts of larvae, including the forty cohorts described above, were assessed for infection from 11 April 2010 to 2 October 2014 by feeding them on mice (Table 3). Those cohorts fed during 2010 to 2012 were 100% positive, while those larval cohorts tested in 2013 and 2014 were 83.3% and 69.2% positive, respectively. This trend suggests that the efficacy of transovarial transmission declined as the colony and its females aged, although the decrease was marginally not significant (*X*^2^ = 3.22, *p* = 0.073).

### 3.4. Prevalence of Infection in Nymphal and Adult O. hermsi

The high prevalence of infection in larval cohorts (Table 3) suggested that with efficient transstadial maintenance of spirochetes, the prevalence of infection in nymphs and adults should also be high, as was shown by the 100% infection in the first F-1 females examined (Table 2). From June 2007 to December 2014 (7.5 years), 103 individual nymphs and adults and 153 mixed pools of nymphs and adults were fed on mice to assess their infection (Table 4). Pools ranged in size from 2 to 55 ticks. The prevalence of infection in both single ticks and pools was high (>90%) during the earlier years but later dropped to 69.2% to 78.4%, suggesting a decline in efficiency in transstadial maintenance of *B. hermsii*. Yet, the maintenance of infectious and transmissible spirochetes in a high a prevalence of ticks for seven years is striking.

### 3.5. Isolation of Spirochetes from Infected Nymphs

On 2 March 2017, seven third-stage nymphs from the second clutch of female F-1D (Table 2) were washed in hydrogen peroxide and ethanol, rinsed in distilled water, triturated and inoculated into individual tubes containing BSK medium, and incubated at 33 °C. Spirochetes grew from five of these ticks, which represented a prevalence of infection of 71.4%. This value was similar to the prevalence of infection of single ticks (75%) assessed by tick feeding three years previously (Table 4). The history of these isolates in the laboratory spanned 8.75 years (21 June 2008 to 2 March 2017) with 10 in vivo passages in ticks that included two transovarial and eight transstadial passages. These results, along with the observations presented above, demonstrate the substantial reservoir capacity of *O. hermsi* to maintain *B. hermsii* for significant periods of time, in this case nearly nine years, without acquiring spirochetes from infected vertebrate hosts.

### 3.6. Microscopic Examination of Single Eggs Versus Same-Clutch Larval Feeding

**Figure 3:**
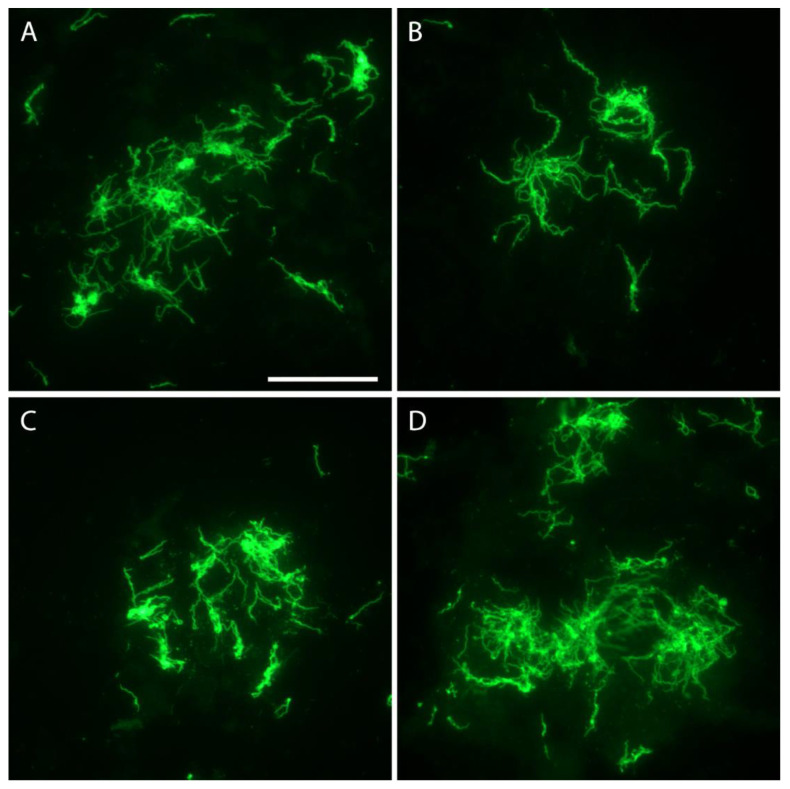
*Borrelia hemsii* aggregates in four eggs (Panels **A**–**D**) from *O. hermsi* female F-1F (Table 5) stained with anti-*B. hermsii* rabbit hyperimmune serum and goat anti-rabbit FITC. Scale bar = 40 µm.

**Figure 4:**
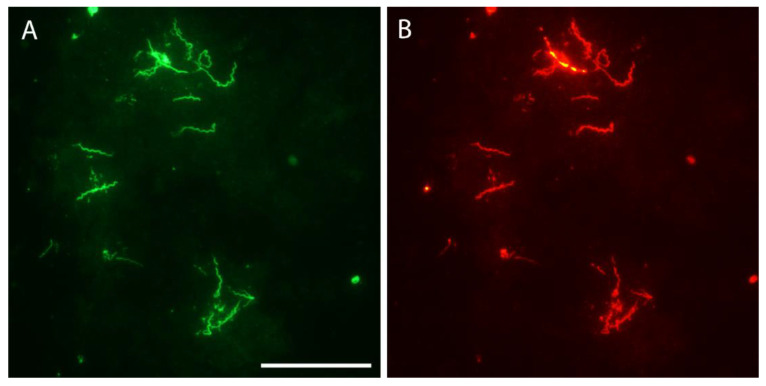
*Borrelia hermsii* in an *O. hermsi* egg stained with anti-*B. hermsii* rabbit hyperimmune serum and goat anti-rabbit FITC (**A**) and stained again with the anti-Vtp mouse monoclonal antibody and goat anti-mouse RITC (**B**). Scale bar = 40 µm.

Immunofluorescent staining was performed to visualize spirochetes in single eggs. In all, 196 eggs were prepared from 13 infected females, but spirochetes were seen in samples from only four ticks (Table 5). Overall, 42 eggs (21.4%; 95% CI, 15.7–27.2%) were infected and the number of *B. hermsii* varied widely from only one or two spirochetes seen per egg to being too numerous to count. The spirochetes stained well with the rabbit anti-*B. hermsii* antibody (Figure 3), as they did when stained again with the mouse monoclonal for the variable tick protein (Vtp) (Figure 4).

Spirochetes were not seen microscopically in eggs sampled from nine of the infected females. Yet, when additional eggs from these same clutches developed into larvae and fed, six of those cohorts infected mice (Table 5). Possibly the number of eggs sampled biased these results as fewer eggs were examined (5 or 12) from the clutches in which no spirochetes were seen compared to the number of larvae fed later (15 to 41). However, the bioassay of feeding larvae on mice was more sensitive than the microscopic examination of eggs to assess transovarial infection of the clutch. Ten of the thirteen clutches (76.9%) were positive by larval feeding, which was significantly greater than only four clutches (30.8%) positive by immunofluorescent staining of the spirochetes in eggs (*X*^2^ = 5.57, *p* < 0.05) (Table 5).

### 3.7. Microscopic Examination of Unfed Larvae

**Figure 5:**
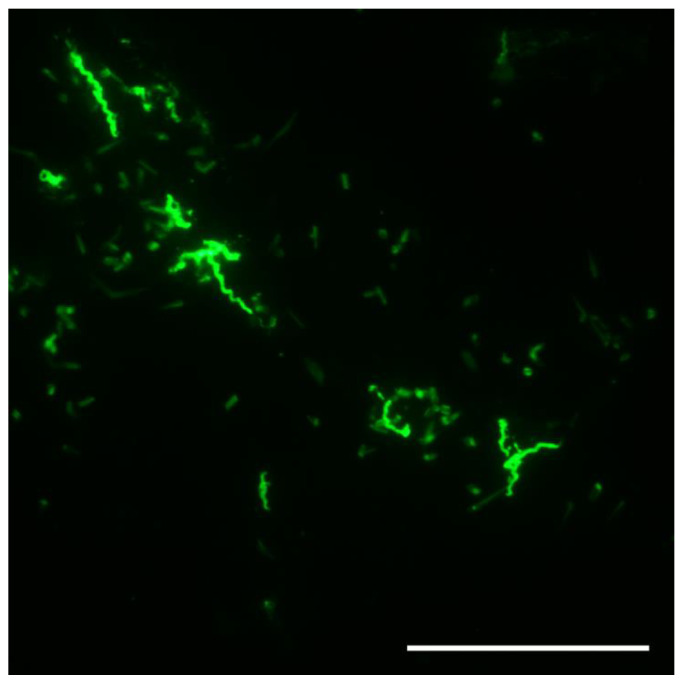
*Borrelia hermsii* in an unfed *O. hermsi* larva stained with anti-Vtp monoclonal antibody and goat anti-mouse FITC. Scale bar = 40 µm.

Because spirochetes in eggs produced Vtp (Figure 4B), immunofluorescent staining with the anti-Vtp antibody was used to visualize *B. hermsii* in unfed larvae produced by five infected females (Table 6). Infected larvae were found in all 5 cohorts, and in total 33 of 60 larvae (55%; 95% CI, 42.4–67.6%) were infected. The spirochetes fluoresced brightly (Figure 5) and were mostly grouped in aggregates that made it impossible to count them visually. Also, the prevalence of infected larvae was significantly greater than the prevalence of infected eggs (*X*^2^ = 24.99, *p* < 0.0001). Clearly, spirochetes were very abundant in unfed larvae and produced Vtp in this first active stage of the tick’s life cycle as well as in eggs.

### 3.8. Tick Infection and Transovarial Passage of a Genomic Group I B. hermsii

Thirteen *O. hermsi* SIS females that fed previously on a mouse spirochetemic with *B. hermsii* DAH together produced about 400 larvae in the single holding tube. Mixed pools of approximately 50 to 60 larvae were fed on each of seven 5-day-old mice, six of which (85.7%) became spirochetemic during the following week. Together, these results demonstrated that *B. hermsii* strains belonging to both genomic groups infecting ticks from different geographic regions and populations of origin could be passed transovarially.

## 4. Discussion

The seminal discoveries that relapsing fever in Africa was caused by a tick-borne spirochete (now known as *Borrelia duttonii*) included the demonstration that progeny from female *O. moubata* may be infectious when fed on an experimental animal [2,4] and that spirochetes were detectable microscopically in tick ovaries and eggs [3,4]. Thus, from these early observations of vertical passage of spirochetes from infected female ticks to progeny via internally infected eggs, the mechanism for true transovarial transmission of borrelia spirochetes by argasid ticks was established. Möllers [21] soon confirmed with multiple laboratory experiments that the progeny from infected female *O. moubata* may be infectious, and Carter [22] followed by examining eggs from a group of naturally infected female *O. moubata*. Six of thirty-two eggs (18.8%) were infected and the number of *B. duttonii* varied from as few as three spirochetes in an entire egg to as many as forty-five spirochetes in one microscopic field of view.

Burgdorfer [5] reviewed additional early work demonstrating transovarial transmission of *B. duttonii* by *O. moubata* and contributed the most thorough investigation of the transmission dynamics involving this spirochete and tick. Included in Burgdorfer’s study were observations pertaining to transovarial transmission, in which 12 of 14 (86%) infected female *O. moubata* produced eggs infected with *B. duttonii*. From these infected females, 211 of 416 eggs (50.7%) were infected, and the percentage of infected eggs from each female ranged from 30% to 70%. Spirochetes were counted in 17 eggs from two females with numbers ranging from 2 to 49 *B. duttonii* per egg. Geigy et al. [23] summarized that 40% to 60% of the progeny of *O. moubata* were infected transovarially, although no experimental details were provided. Aeschlimann [24] observed a similar prevalence of transovarial transmission of *B. duttonii* by *O. moubata* with 18 of 35 (51.4%) infected females passing spirochetes to their progeny. From the 18 infected females, 349 of 709 (49.2%) of the “descendants” were infected.

These early studies helped define how transovarial transmission is described and quantified [25], which is by (1) the prevalence or percentage of infected females that pass spirochetes to any of their progeny, and (2) the percentage of progeny infected by single infected female ticks (filial infection prevalence). The product of these two variables, the vertical transmission rate (VTR) [26] provides a better overall assessment of the contribution that vertical passage makes at the population level. The number of spirochetes internalized by single eggs from the female also has the potential to impact the role of transovarial transmission in the maintenance cycle. Thus, those vector species that infect more of their offspring, produce a higher prevalence of filial infection, and provide a greater inoculum per egg would have the greatest potential to maintain a spirochete population in nature via transovarial transmission.

Considerable early work by Russian epidemiologists and vector biologists was directed at argasid ticks and spirochetes causing relapsing fever in Asia. Two important English translations of Russian monographs include discussions of *Ornithodoros* species and their ability to transovarially infect their progeny with borreliae [27,28]. While the taxonomic names for ticks and spirochetes have varied between Russian and American scientists, numerous studies show that what is now accepted as *Borrelia persica* is commonly passed transovarially by it vector *Ornithodoros tholozani*. Experimental studies in Egypt also demonstrated efficient transovarial transmission of *Borrelia crocidurae* by *Ornithodoros erraticus* [29] and the fowl spirochete *Borrelia anserina* by two species of *Argas* ticks [30].

In North America, only a few species of soft ticks and their respective spirochetes have been studied for transovarial transmission. *Ornithodoros turicata* is the specific vector of the relapsing fever spirochete *Borrelia turicatae* [10]. Studies demonstrating transovarial transmission for this tick and spirochete are relatively few and date back to the 1930s. Brumpt [31] included in his brief description and naming *Borrelia turicatae* (=*Spirochaeta turicatae*) a summary stating that infected female *O. turicata* transmitted spirochetes to 40% of the progeny, and soon followed that the observations were based on 4 of 10 white mice being infected by ticks feeding on them [32]. Francis [33,34] demonstrated transovarial transmission by infecting two mice with subcutaneous inoculations of unfed larvae from two female *O. turicata* collected in Texas. Francis [34] also infected three mice by feeding large groups of larvae (75, 175, and 270 ticks per group) from females infected as nymphs in the laboratory. Two larvae also fed on Francis, which resulted in him becoming infected and developing relapsing fever. Davis [35] also reported a human laboratory-acquired infection of *B. turicatae* caused by the bite of a single transovarially infected *O. turicata* larva, further demonstrating the potential hazard when working with very small ornithodorine larvae (Figure 2) infected transovarially with relapsing fever spirochetes. Results presented herein demonstrate that caution and proper containment must also be taken by investigators working in the laboratory with *O. hermsi* larvae coming from females infected with *B. hermsii*.

Davis [36] performed a multiyear study with a colony of infected *O. turicata* that began with a single female tick that originated from a naturally infected nymph, as was the colony of *O. hermsi* described in the present work. Five generations of ticks were followed over six years by feeding some ticks in groups but mostly single ticks on mice to assess their prevalence of infection with *B. turicatae*. Once a new generation of adult ticks was attained, a single infected female was chosen and the prevalence of its offspring infected transovarially was assessed. As this new cohort of larvae developed through their multiple nymphal stages to adults, another new infected female was chosen and followed. Thus, feeding progeny from the first oviposition of five infected females from five successive generations showed that 35%, 96%, 100%, 47%, and 98%, respectively, of the larval ticks were infected by transovarial transmission. These results led Davis [36] to conclude “that the tick itself may be a more efficient spirochetal reservoir than the rodent host.” Yet oddly, Davis [37] never observed *Borrelia parkeri* to be passed transovarially by it vector *Ornithodoros parkeri* in spite of the close phylogenetic relationship of *O. parkeri* to *O. turicata* [38,39] and *B. parkeri* to *B. turicatae* [40]. Because the efforts that Davis made to address transovarial transmission of *B. parkeri* by *O. parkeri* are unknown, future work investigating vertical passage of these spirochetes by *O. parkeri* is warranted.

Transovarial transmission has been demonstrated for *Borrelia coriaceae* by its tick vector, *Ornithodoros coriaceus*, although the frequency of its occurrence may be rare. Lane et al. [41] and Lane and Manweiler [42] employed immunofluorscent staining to detect spirochetes in fixed smears of field-collected *O. coriaceus* larvae from California and together, found only 10 of 1403 (0.71%) ticks infected. One hundred larvae from each of nine infected females were also examined with just one female producing infected larvae (14% infected), for an overall prevalence of infection of only 1.56%. However, as shown herein for *B. hermsii* (Table 5), immunofluorescent stains and microscopy may not be the most sensitive method for determining infections.

Only two early studies examined transovarial transmission of *B. hermsii* by *O. hermsi*. Herms and Wheeler [12] and Wheeler [13] presented observations of the same experiments that showed only 2 of 672 larvae produced by 7 female *O. hermsi* were infected, yielding a prevalence of infection by transovarial transmission of only 0.29%. Longanecker [14] found three of seven infected female *O. hermsi* produced infected larvae, which when fed on mice in groups of ten ticks, yielded an estimated minimum prevalence of infection of 5.3%. Davis [35,43] also mentioned that infected female *O. hermsi* involved with various transmission studies subsequently produced infected progeny but additional information was not provided. Finally, Burgdorfer and Varma [25] included a personal communication from Gordon Davis stating that he observed “filial infection rates, up to 12 per cent” for *O. hermsi* infected with *B. hermsii* but the data were never published. Thus, the longstanding claim that *O. hermsi* rarely passes *B. hermsii* transovarially has been predicated on only two short-term studies using a small number of female ticks that showed if *B. hermsii* was maternally transferred to progeny, only 0.29% to 5.3% of the larvae were infected. In contrast, the colony described herein demonstrated that *B. hermsii* can be passed transovarially by a high proportion of infected female *O. hermsi*, and single infected females may infect a large proportion of their progeny. This vertical passage of *B. hermsii* was maintained over several years without acquisition of spirochetes from vertebrate hosts, as described for *O. moubata* infected with *B. duttonii* [24] and *O. turicata* infected with *B. turicatae* [36]. Additionally, our infected F-1 *O. hermsi* females (Table 2) and the number of infected larvae from a subset of these females (Table 5) provide an estimated overall vertical transmission rate (VTR) of 48.9%. Thus, if our colony fairly represents the population of *O. hermsi* at Mt. Wilson (where the founding female originated) the ticks there would produce nearly 50% of their next generation infected with *B. hermsii*. This estimate is far greater than our calculation based on the only other minimal available data provided by Longanecker [14], which is just 3.2%. Thus, our results show that infected *O. hermsi* may produce a high prevalence of next-generation ticks infected with *B. hermsii* in an endemic focus of tick-borne relapsing fever.

The multigenerational propagation of a high prevalence of infected ticks originating from the single founding *O. hermsi* female without spirochete amplification in mice clearly implicates *B. hermsii* replicated in ticks. Koch [3,4] suggested from his seminal observations that *B. duttonii* replicated in the ovary and eggs of *O. moubata*, and noted that freshly laid eggs, if infected, had single and dispersed spirochetes but as eggs matured, spirochetes were seen more often in groups of “piles and braids”, as observed in some *O. hermsi* eggs infected with *B. hermsii* (Figure 3). This led Koch to conclude the following: “So it seems that they [spirochetes] continue to multiply in the eggs as well” [4]. The bloodmeal of female ticks is converted to egg yolk through the process of vitellogenesis with the precursors of the vitellin proteins produced in the tick’s fat body and possibly the midgut [44] and dispersed via the hemolymph to the developing oocytes. Mature tick eggs are nutrient-rich to support the growth of the embryo, with the yolk composed of multiple proteins, lipids, and carbohydrates [44,45]. This complex mixture of nutrients in the egg yolk also appears suitable to support the growth of spirochetes and certainly deserves further investigation. The size of ticks influences the amount of blood ingested and the number of eggs produced—larger species ingest more blood and produce more eggs per oviposition [28]. *Ornithodoros hermsi* is one of the smaller tick vectors of relapsing fever spirochetes [38,46] and females average only about 17 to 22 µL of blood per feeding [17]. While the size of the bloodmeal was not measured in the work presented herein, the average number of larvae produced per bloodmeal was only 35, and ranged from 5 to 71 larvae in 45 cohorts monitored. Thus, the contribution of spirochetes to be passed to the next generation of ticks might be less due to the low fecundity of this small tick compared to larger argasid vector species. Spirochetes were counted in eggs with little or no embryonic development, eggs further along in their maturation, and unfed larvae (Figure 2). There was a wide range in the number of spirochetes observed by immunofluorescent staining of single eggs. In the 15 infected eggs examined from female F-1C (Table 5), the 12 infected eggs with no or minor embryonic development contained 2–75 spirochetes ($\bar{x}$ = 16.4) while the three eggs with obvious embryos present contained 15–128 spirochetes ($\bar{x}$ = 62). This sample is small with overlapping numbers of spirochetes in the two groups, and the number of spirochetes inoculating each egg in the female tick prior to oviposition was not known. However, these numbers and the greater prevalence of larvae with microscopically detectable spirochetes (55%) compared to eggs (21.4%), together support the notion that *B. hermsii* replicated in *O. hermsi* eggs.

The *B. hermsii* maintained transstadially and transovarially in the *O. hermsi*-colonized ticks produced Vtp in eggs and larvae. In the present-day life cycle of *B. hermsii* with spirochetes alternating between ticks and mammals, Vtp is produced by spirochetes while persistently residing in the tick’s salivary glands [18,47]. Following transmission by tick bite to mice, *B. hermsii* replaces Vtp with one of the variable major proteins (Vmps) [18,47] as the program of multiphasic antigenic variation proceeds to allow spirochetes to sequentially evade the host’s antibody response [48,49]. These repeated spirochetemias increase their opportunity for horizontal acquisition by naïve ticks [50]. Once back in ticks, the *B. hermsii* population replaces the Vmp with Vtp as the spirochetes again colonize the salivary glands. Thus, Vtp is a borrelia tick-specific protein. Given the concept that spirochetes evolved first as symbionts of argasid ticks [7], Vtp might represent a major surface protein made by these spirochetes during their symbiotic life in ticks before becoming tick-borne parasites of vertebrates. Vtp is immunogenic in mice [51,52], and immunological pressure from previously infected vertebrate hosts during the early expansion to include vertebrates may have driven the polymorphism of this locus seen today. Deduced amino acid sequences of the *vtp* gene from 79 *B. hermsii* samples collected from many locations of western North America segregate into nine groups, designated Types 1–9 [11,19,53], and multiple strains of *B. hermsii* containing different Vtp types occur sympatrically in the same endemic focus [11,54]. Within each Vtp group the amino acid sequences are either identical or vary little (<8%), but between groups the sequences share only 58.5% to 74.2% identity [11,19,53], making the proteins of each group antigenically distinct from the others. Mice immunized with purified recombinant Vtp of one type are protected from infection with *B. hermsii* when challenged with ticks infected with the homologus Vtp-producing strain but not from a heterologous strain producing a Vtp with only 69.9% identity [51]. Additionally, *B. hermsii* that is unable to switch from Vtp to its Vmp system of antigenic variation following transmission by tick bite (a phenotype that may represent the ancestral pattern of vertebrate infection) is rapidly cleared with no subsequent relapses to repopulate the blood [47]. Thus, the evolution of a mechanism to replace Vtp with a series of antigenically distinct surface proteins to sequentially evade the host’s antibody response was an essential step for these bacteria to cyclically prolong spirochetemias in the peripheral blood for their acquisition by small, fast-feeding ticks.

Interest in transovarial transmission of borreliae has had a recent resurgence, not with argasid tick-borne spirochetes but with the discovery of the hard tick-transmitted relapsing fever spirochete *Borrelia miyamotoi* infecting *Ixodes persulcatus* in Japan [55,56]. However, another ixodid tick-borne borrelia, *Borrelia theileri*, was discovered by Arnold Theiler in South African cattle over one hundred years ago [57,58], and which Theiler [59,60] showed was passed transovarially by *Rhipicephalus* (*Boophilus) decoloratus* and *Rhipicephalus (Rhipicephalus) evertsi*. Yet interest in transovarial transmission of *B. theileri* has received only little attention [61,62], probably because this spirochete is not a human pathogen and causes only minor disease in cattle and other large domestic animals. The history and emerging public health significance of *B. miyamotoi* were reviewed, in part, elsewhere [26,63,64], as was this spirochete’s mistaken identity as *Borrelia* (*=Borreliella*) *burgdorferi* and other Lyme disease spirochetes in field-collected, unfed larval *Ixodes* ticks prior to its discovery in 1995 [65,66]. In North America, Scoles and coworkers [67] were first to find *B. miyamotoi* in the northeastern United States while examining *Ixodes scapularis* for spirochete infection in larvae produced by field-collected female ticks fed in the laboratory. Larval groups of 50 ticks each from clutches produced by 52 females were tested by PCR, with two clutches (3.8%) infected with *B. miyamotoi.* Fifty more larvae were tested individually from the two infected clutches, which demonstrated prevalences of filial infection of 6% and 73%. Rollend et al. [66] examined larval progeny from many more field-collected *I. scapularis* females from Connecticut using the same methods as Scoles et al. [67] and found, during an 11-year period, that 19 of 1214 clutches (1.6%) were infected with *B. miyamotoi*. In neither of these studies were the ovipositing females tested for infection, so the efficiency of transovarial transmission by infected females could not be determined. Han et al. [68] collected engorged female *I. scapularis* from carcasses of white-tailed deer (*Odocoileus virginianus*) and allowed these ticks to oviposit in the laboratory. During two hunting seasons in 2015 and 2016 in four midwest and eastern States, 33 of 1263 (2.6%) females were infected with *B. miyamotoi*, and of those females infected, 11 ticks laid eggs with 10 of the clutches (90.9%) infected transovarially. The prevalence of filial infection in nine of the clutches ranged from 36% to 100% ($\bar{x}$ = 84.4%). Lynn et al. [69] tested *I. scapularis* nymphs originating as larvae from two field-collected females from Minnesota and Connecticut that were fed in the laboratory, yielding prevalences of filial infection of 80% and 88%, respectively. Most recently, Keesing et al. [70] tested *I. scapularis* larvae collected by flagging in New York, with 8 of 216 pools (10 ticks per pool) infected with *B. miyamotoi* that provided an estimated prevalence of infection of only 0.4%. In Europe, Richter et al. [65] tested field-collected *Ixodes ricinus* larvae from the Czech Republic; 1 of 156 (0.64%) ticks was infected as were 6 of 99 pools (6.1%) (5 ticks per pool). Larvae were also tested from engorged female *I. ricinus* collected from dogs in Germany; 16 of 510 pools (3.1%) (5 ticks per pool) were infected, and from the 16 positive pools, 126 of 136 larvae (92.6%) were infected. In The Netherlands, van Duijvendijk et al. [71] fed field-collected *I. ricinus* larvae on mice in the laboratory (some of which became infected) and then tested these ticks after they molted to nymphs; 29 of 1456 (1.9%) were infected with *B. miyamotoi*.

Clearly, those hard tick-transmitted spirochetes that group together phylogenetically in their own clade of relapsing fever borreliae (*B. theileri*, *B. miyamotoi*, and *B. lonestari* [8]) can be passed transovarially by infected females to their progeny. Yet, examining questing larvae from the field that have a very low prevalence of infection [70,72], poses the possibility that these ticks became infected by partial, interrupted feedings on infected hosts before being collected and examined. Such ticks would be falsely positive if considering transovarial passage as the source of infection. However, the results reviewed above for studies with unfed larvae produced by females in the laboratory are unequivocal [65,66,67,68,69].

The attention to transovarial transmission of *B. miyamotoi* has increased since the recognition of this spirochete as a human pathogen [73,74,75,76], and the possibility that transovarially infected larvae may pose a risk for human infections. Breuner et al. [77] supported this notion by demonstrating a single transovarially infected *I. scapularis* larva can infect a mouse while feeding. Research on transovarial transmission of *B. miyamotoi* has been facilitated by several factors, including (1) the numerous investigators experienced in working with *Ixodes* species because of the role these ticks play in Lyme disease, (2) the relative ease of collecting these ticks while they quest on vegetation compared to the secretive and nidicolous behavior of *Ornithodoros* species [26,78], and (3) by adapting existing PCR assays to rapidly examine large numbers of ticks. The recent development of an improved culture medium to grow *B. miyamotoi*, which was used to isolate the spirochete from transovarially-infected *I. scapularis* nymphs [79], is also an important advance for such studies.

Natural populations of ticks in the *I. ricinus* complex have low prevalences of infection with *B. miyamotoi*, yet those females infected may pass spirochetes on to a large proportion of their progeny, as cited above. Given that fully engorged *I. scapularis* and *I. ricinus* females may lay 1000 to 3000 eggs during their one-only oviposition [28,80], those few females that are infected may have a significant impact on seeding local foci with *B. miyamotoi*-infected larvae. Recall that the founding *O. hermsi* female described herein produced only 195 larvae (189 that fed) from nine bloodmeals over four years. Randolph [81] constructed a model predicting that transovarial transmission of *B. burgdorferi* (even with low prevalences of vertical passage and high mortality at each stage of the ixodid tick’s life cycle) would contribute significantly to the maintenance of the spirochete in nature. This conclusion based on the model could be applied to *B. miyamotoi* instead, as the observations and data used were almost certainly based on the presence of this spirochete in larval ticks and not *B. burgdorferi*, which is not passed transovarially [65,66]. Hopefully, investigators will establish colonies of *Ixodes* species infected with *B. miyamotoi* to gain a better understanding of the potential contribution that only transovarial and transstadial passage provide for the maintenance of this spirochete over successive generations of ticks without spirochete acquisition from infected vertebrates. Such infected colonies would also support studies on the phenotypic expression of surface proteins as *B. miyamotoi* alternates infections between ticks and vertebrates throughout its life cycle, as has been shown for *B. hermsii* [18,47,53].

## 5. Conclusions

The observations presented herein demonstrate that *B. hermsii* can be efficiently passed transovarially and transstadially by *O. hermsi* without amplification in vertebrate hosts. The perpetuation of these spirochetes in ticks alone for nearly nine years also demonstrates the potential for *O. hermsi* to serve not only as the specific vector of *B. hermsii* but as its potential reservoir as well. A recent model predicted the minimum number of relapses required with different combinations of vertebrate host species for *B. hermsii* to achieve an *R_o_* ≥ 1 and maintain endemicity [82]. Future models will benefit by adding a transovarial transmission component to better understand the contribution of vertical passage of *B. hermsii* by infected female *O. hermsi* to maintain an endemic focus. The infection and replication of *B. hermsii* in yolk-filled eggs of *O. hermsii* and the potential competition between spirochetes and the developing embryo are intriguing topics for future investigations. A thorough examination of spirochetal growth and nutrient composition of *O. hermsi* eggs might also offer insights into improving the efficacy of in vitro cultivation of these borreliae directly from their natural biological sources.

## Figures and Tables

**Table 1:** *Ornithodoros hermsi* founding female’s feeding history, oral transmission, and production of infected offspring after each bloodmeal.

Founding Female History			Larval Assays			Nymphal Assays	
Feeding	Date Fed	Transmission to Mouse	DF ^a^	Number Fed	Transmission to Mouse	Stage Fed ^b^	Transmission to Mouse
1st	4 March 2009	ND ^c^	2/2	33	ND	N^2^	+ ^d^
2nd	16 December 2009	ND		25	+	N^2^, N^3^	+,+
3rd	11 April 2010	+		35	ND	N^1^	+
4th	27 August 2010	ND		18	ND	N^2^, N^3^	+,+
5th	27 June 2011	ND	1/1	45	+	N^1^, N^2^, N^3^	+,+,+
6th	18 December 2011	+	1/1	27	+	N^1^, N^2^, N^3^	+,+,+
7th	27 April 2012	+	2/2	6	+	N^1^	+
8th	8 November 2012	- ^e^		0 ^f^	NA ^g^	0	NA
9th	16 April 2013 ^h^	-		0	NA	0	NA

^a^ DF = number infected /number unfed larvae examined by dark-field microscopic examination; ^b^ N^1^ = first stage nymphs, N^2^ = second stage nymphs, N^3^ = third stage nymphs; ^c^ ND = transmission to mice by feeding not determined; ^d^ + = positive by transmission by the respective stages to mice; ^e^ - = no spirochetemia detected; ^f^ 0 = no eggs produced after bloodmeal; ^g^ NA = not applicable; ^h^ founding female found dead 27 September 2013.

**Table 2:** *Borrelia hermsii* infection in F-1 female *O. hermsi* and larval cohorts assayed by transmission to mice.

Female ID	Clutch Origin ^a^	Date Female First Fed	Infection by Feeding	Larval Cohorts Infected ^b,c^
F-1A	First	25 January 2011	+	2/2
F-1B	First	“	+	4/5
F-1C	First	“	+	3/3
F-1D	First	6 August 2011	+	3/3
F-1E	First	“	+	3/3
F-1F	First	“	+	1/1
F-1G	First	“	-	2/2
F-1H	First	1 September 2011	+	None Produced
F-II	First	“	+	4/4
F-1J	Second	10 November 2012	+	2/2
F-1K	Second	“	+	1/2
F-1L	Second	“	+	0/2
F-1M	Second	“	+	2/2
F-1N	Second	“	+	1/1
F-1O	Second	“	+	0/2
F-1P	Second	16 April 2013	+	1/2
F-1Q	Second	“	+	1/1
F-1R	Second	11 December 2012	+	2/2
F-1S	Second	1 March 2013	+	1/1
Totals			18/19	33/40 (82.5%)

^a^ First = first clutch of founding female, Second = second clutch of founding female; ^b^ number infected cohorts/number cohorts tested; ^c^ larval cohorts fed 17 December 2011–2 October 2014. “ = Same as date above

**Table 3:** Assessment of transovarial infection of *O. hermsi* larvae with *B. hermsii* by feeding larval cohorts on mice at different times after tick colonization.

Dates Larvae Fed	Source Females ^a^	Larval Cohorts Fed	Larvae Per Cohort	Larval Cohorts Infected	Percentage Cohorts Infected
April 2010–December 2011	4	5	25–60	5	100%
April–November 2012	7	9	6–70	9	100%
July–September 2013	18	18	5–70	15	83.3%
October 2014	13	13	14–57	9	69.2%
Total	20	45 ^b^		38	84.4%

^a^ number of female ticks producing the larval cohorts tested for infection; ^b^ includes the 40 cohorts in Table 2 plus 4 cohorts from the founding female and 1 cohort from a second generation (F-2) female.

**Table 4:** Assessment of nymphal and adult *O. hermsi* infected with *B. hermsii* by feeding single and pooled ticks on mice at different times after tick colonization.

	Single Ticks Fed			Pooled Ticks Fed		
Dates Ticks Fed	Number Ticks Infected	Number Ticks Fed	% Infected	Number Pools Infected	Number Pools Fed	% Infected
June 2007–December 2011	43	47	91.5%	14	15	93.3%
April–December 2012	24	26	92.3%	28	30	93.3%
March–December 2013	18	26	69.2%	55	71	77.5%
April–December 2014	3	4	75.0%	29	37	78.4%
Total	88	103 ^a^	85.4%	126	153 ^b^	82.4%

^a^ 12 nymphs, 42 males, 49 females; ^b^ 117 pools of nymphs (2–55 ticks), 10 pools of nymphs and adults (2–15 ticks), 26 pools of adults (2–7 ticks).

**Table 5:** Examination of eggs from infected *Ornithodoros hermsi* females by immunofluorescent microscopy and same-clutch larval feeding on mice.

Female ID	Eggs Infected/Eggs Examined	Average Number Spirochetes Per Egg (Range)	Number of Larvae Fed in Cohort	Larval Cohort Transmission ^a^
F-1C	15/38	25.5 (2–128)	23	+
F-1E	13/24	43.0 (1–110)	5	+
F-1F	11/12	97.0 ^b^ (15–TNC)	29	+
F-1S	3/24	3.7 (1–9)	8	+
F-1G	0/12	0	17	+
F-1M	0/12	0	41	+
F-1P	0/12	0	15	+
F-1Q	0/12	0	34	+
F-1R	0/12	0	35	+
F-2A	0/5	0	27	+
F-1B	0/6	0	9	-
F-1L	0/12	0	22	-
F-1O	0/15	0	18	-
Totals	42/196 (21.4%)			

^a^ These 13 larval cohort feedings are included in the totals presented in Table 3; ^b^ This average is based on 6 of the 11 infected eggs in which the spirochetes were not too numerous to count (TNC).

**Table 6:** *Ornithodoros hermsi* larvae infected with *B. hermsii* by transovarial transmission determined by immunofluorescence microscopy and staining for the variable tick protein (Vtp).

Female ^a^	Number Larvae Infected/ Number Larvae Examined ^b^	Percentage Larvae Infected
F-1F	8/12	66.7%
F-1I	9/12	75%
F-1J	2/12	16.7%
F-1Q	10/12	83.3%
F-1T	4/12	33.3%
Total	33/60	55%

^a^ Females fed 25 September 2015; ^b^ Larvae examined 14–17 March 2016.
